# The Significance of N6-Methyladenosine RNA Methylation in Regulating the Hepatitis B Virus Life Cycle

**DOI:** 10.4014/jmb.2309.09013

**Published:** 2023-10-31

**Authors:** Jae-Su Moon, Wooseong Lee, Yong-Hee Cho, Yonghyo Kim, Geon-Woo Kim

**Affiliations:** 1 Division of Infectious Diseases, Department of Medicine, University of California, San Diego, La Jolla, CA 92093, USA; 2 Center for Convergent Research of Emerging virus Infection, Korea Research Institute of Chemical Technology (KRICT), Daejeon 34114, Republic of Korea; 3 Data Convergence Drug Research Center, Therapeutics and Biotechnology Division, Korea Research Institute of Chemical Technology (KRICT), Daejeon 34114, Republic of Korea; 4 Department of Medical Chemistry and Pharmacology, University of Science and Technology (UST), Daejeon 34113, Republic of Korea; 5 Department of Microbiology and Molecular Biology, Chungnam National University, Daejeon 34134, Republic of Korea

**Keywords:** Hepatitis B virus, N6-methyladenosine RNA methylation, HBV life cycle, innate immunity, HBV chronic infection, hepatocellular carcinoma

## Abstract

N6-methyladenosine (m6A) RNA methylation has recently emerged as a significant co-transcriptional modification involved in regulating various RNA functions. It plays a vital function in numerous biological processes. Enzymes referred to as m6A methyltransferases, such as the methyltransferaselike (METTL) 3-METTL14-Wilms tumor 1 (WT1)-associated protein (WTAP) complex, are responsible for adding m6A modifications, while m6A demethylases, including fat mass and obesity-associated protein (FTO) and alkB homolog 5 (ALKBH5), can remove m6A methylation. The functions of m6A-methylated RNA are regulated through the recognition and interaction of m6A reader proteins. Recent research has shown that m6A methylation takes place at multiple sites within hepatitis B virus (HBV) RNAs, and the location of these modifications can differentially impact the HBV infection. The addition of m6A modifications to HBV RNA can influence its stability and translation, thereby affecting viral replication and pathogenesis. Furthermore, HBV infection can also alter the m6A modification pattern of host RNA, indicating the virus's ability to manipulate host cellular processes, including m6A modification. This manipulation aids in establishing chronic infection, promoting liver disease, and contributing to pathogenesis. A comprehensive understanding of the functional roles of m6A modification during HBV infection is crucial for developing innovative approaches to combat HBV-mediated liver disease. In this review, we explore the functions of m6A modification in HBV replication and its impact on the development of liver disease.

## Introduction

**Fig. 1:**
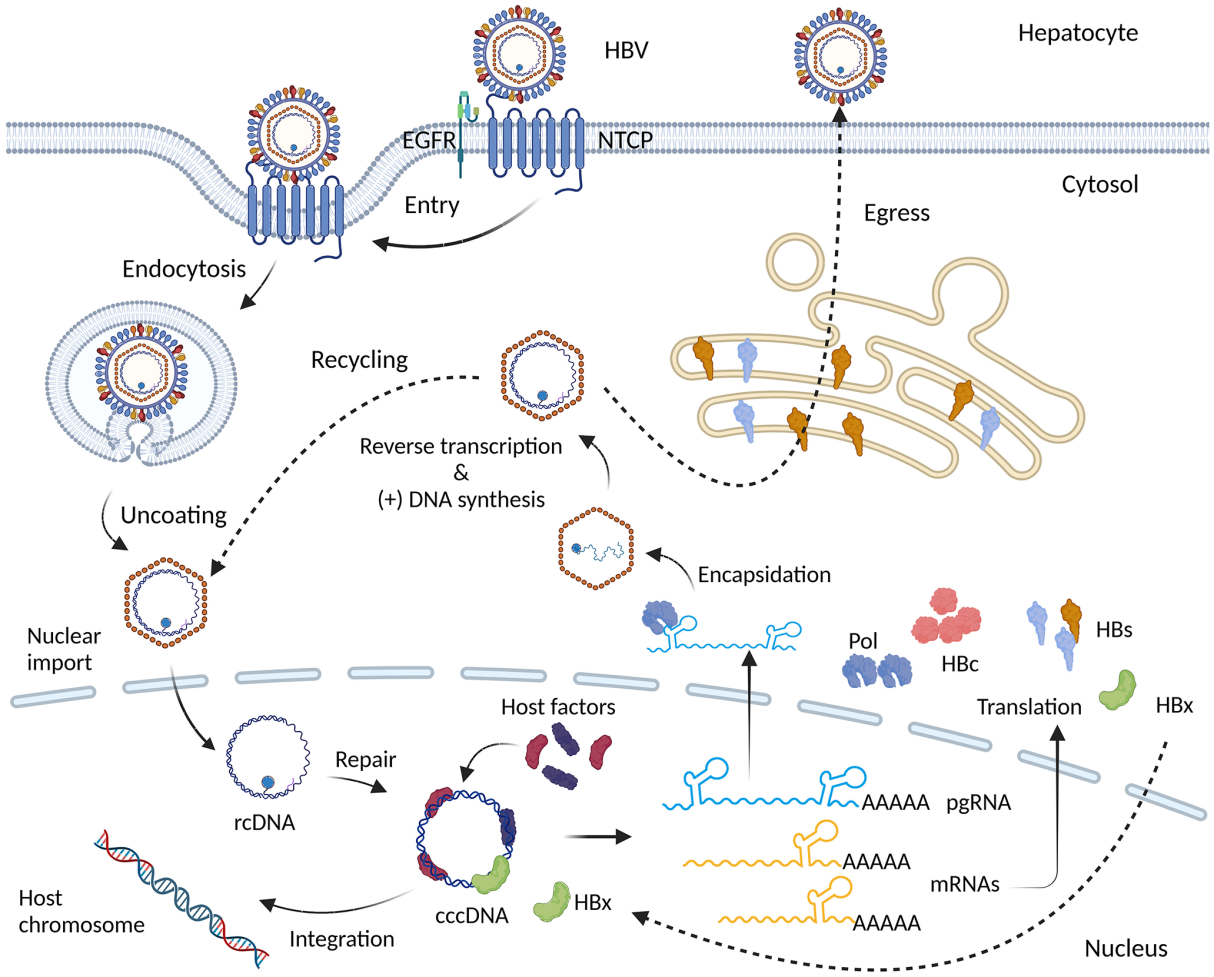
Overview of the Hepatitis B Virus (HBV) Life Cycle. HBV enters human liver hepatocytes through the Na+- taurocholate cotransporting polypeptide (NTCP) receptor and the epidermal growth factor receptor (EGFR) co-receptor. After entering the cells, the viral particle undergoes uncoating on the nuclear membrane. Its genetic material (rcDNA) is then transported into the nucleus, where rcDNA is converted to cccDNA, serving as the template for HBV mRNA transcription. The Viral mRNAs are subsequently transported to the cytoplasm for protein translation. Viral polymerase interacts with 5’ epsilon structure of pgRNA , promoting nucleocapsid assembly. Within the core particles, the (-) strand DNA is generated by reverse transcription, followed by pgRNA degradation and the synthesis of the (+) strand DNA, resulting in rcDNA formation. Mature core particles have two potential fates: they can be enveloped to form virions for release, or they can be transported to the nucleus to produce additional cccDNA. Additionally, double-stranded linear DNA, which is an abnormal replication product of pgRNA, is the preferred template for integration into the host chromosomal DNA.

Hepatitis B virus (HBV) is a member of the *Hepadnaviridae* family and possesses the 3.2 kb partially double-stranded relaxed circular DNA (rcDNA) genome [1]. HBV infection triggers chronic hepatitis and leads to liver cirrhosis, representing a significant risk factor for the development of hepatocellular carcinoma (HCC) [2]. HBV particle enters hepatocytes by binding to the Na/taurocholate co-transporting polypeptide (NTCP) receptor, facilitated by the epidermal growth factor receptor (EGFR) as co-receptor (Fig. 1) [3, 4]. After infection, HBV nucleocapsid moves to the nucleus membrane and releases rcDNA into the nucleus [5]. In the nucleus, rcDNA genome is converted to a covalently closed circular DNA (cccDNA) by viral polymerase and host factors. Viral RNAs are transcribed by cellular polymerase II using cccDNA as a template. All viral RNAs originated from distinct transcription initiation sites on the HBV genome but share a common poly A site at their ends [5]. These HBV RNAs encode both structural and nonstructural viral proteins, including surface (HBs), precore or ‘e’ (HBe), and core (HBc) antigen, polymerase, and X (HBx) proteins. Viral polymerase (P) recognizes pregenomic RNA (pgRNA) to initiate encapsidation with core proteins [1, 5]. Following nucleocapsid assembly, the reverse transcription enzymatic function of viral polymerase synthesizes negative strand viral DNA as the template of pgRNA. Subsequently, a positive strand of viral DNA is generated from negative strand viral DNA [1, 5]. The nucleocapsids containing the viral genome are either recycled to amplify and sustain the cccDNA or matured with viral surface proteins for secretion outside the cells [1, 5].

**Fig. 2:**
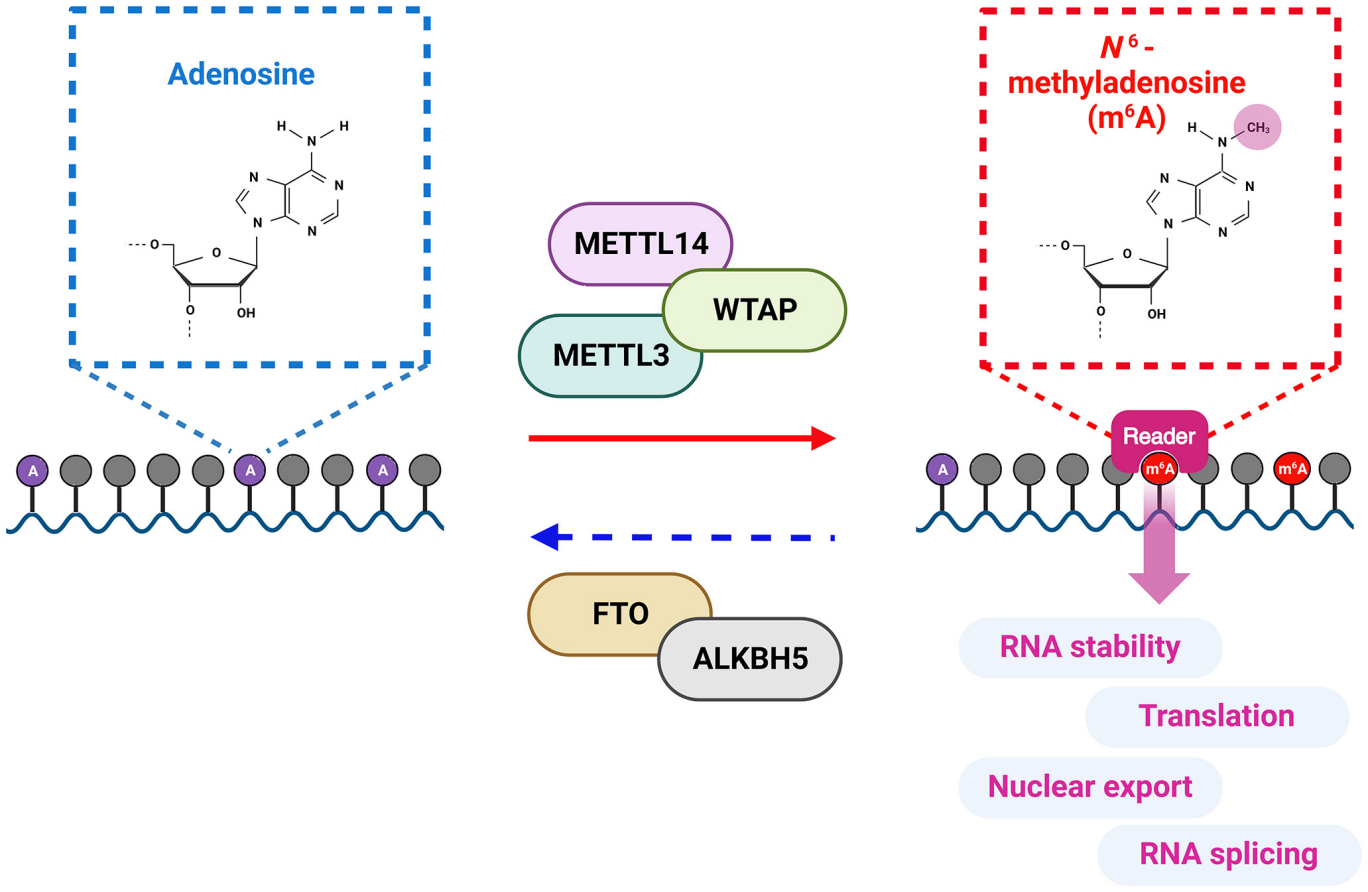
Cellular m6A machinery: Writers, Erasers, and Readers. The cellular m6A machinery involves three main components: Writers: The writer complex is composed of core subunits METTL3 and METTL14, along with WATP as an additional adaptor protein. Erasers: Known m6A erasers include FTO and ALKBH5, responsible for demethylating m6A modifications. Readers: The readers of m6A-modified RNAs consist of YTH-domain-containing proteins (YTHDF1-3) and YTH N6-methyladenosine binding proteins (YTHDC1-2) that directly bind m6A-methylated RNAs and regulate the dynamics of their target RNAs.

In the 1970s, N6-methyladenosine (m6A) methylation was discovered, but technical limitations at that time prevented the identification of individual m6A locations in RNA [6]. In recent times, technological progress, including high-throughput sequencing, has enabled the identification of m6A distribution within the cellular transcriptome, as well as individual-specific m6A sites in RNA [7]. m6A methylation, which involves adding of a methyl group to the adenosine base at position nitrogen 6, is the most extensively studied and common internal modification in cellular RNAs [7, 8]. This modification is associated with essential biological processes, including immune response, cell differentiation, circadian clock regulation, sex determination, stress responses, and carcinogenesis [8]. m6A modifications are found in more than 25% of mammalian RNAs, occurring within the consensus m6A motif (DRACH; D = A, G, or U; R = G or A; H = A, C, or U) [7]. However, all DRACH motifs are not m6A methylated. Generally, these modifications are located in the 5' and 3'-untranslated region (UTR) and near the stop codon [7]. m6A modification is a reversible process facilitated by m6A methyltransferases and demethylases. The cellular m6A methyltransferase machinery, including methyltransferase-like 3 (METTL3), METTL14, and WT1-associated protein (WTAP), adds m6A methylation to cellular and viral RNAs (Fig. 2) [9]. Conversely, m6A modification of RNAs can be removed by m6A demethylases, such as AlkB homolog 5 (ALKBH5) and Fat mass and obesity-associated protein (FTO) [10, 11]. Various m6A reader proteins, belonging to YT521-B homology (YTH) domain family proteins (YTHDF1/2/3) and YTH N6-methyladenosin RNA binding protein (YTHDC1/2), regulate the functions of m6A-modified RNAs [12]. YTHDF3 interacts with m6A methylated RNA and recruits the YTHDF1 or 2 protein onto its target RNA [13]. YTHDF1 promotes the translation of m6A-methylated mRNA, facilitating the process of protein synthesis, whereas YTHDF2 is involved in the degradation of its target m6A-methylated RNAs, leading to the decay of mRNA molecules [14, 15]. The degradation of m6A methylated RNA by YTHDF2 is mediated by its interaction with the carbon catabolite repression-negative on TATA-less (CCR4-NOT) deadenylase complex, due to the lack of RNase activity of YTHDF2 [15]. YTHDC1 is involved in mRNA nuclear export in collaboration with nuclear RNA export factor 1 (NXF1), and also plays a role in RNA splicing [16]. YTHDC2 contains RNA helicase domain and enhances the translation of m6A-modified mRNA by interacting with a small ribosomal subunit [17]. The RNA helicase activity of YTHDC2 is required to unwind mRNA secondary structures, thereby facilitating mRNA translation [18]. Therefore, the functions of m6A-methylated RNA are epigenetically regulated through interactions with a variety of m6A reader proteins.

The multifaceted roles of m6A methylation within the viral RNA during infection by RNA viruses as well as DNA viruses have garnered attention and have been underscored in various studies [19-28]. m6A RNA methylation intricately regulates viral infections and affects the outcomes of host-virus interactions. By Adding m6A to the viral RNA genome and transcript, it directly regulates viral RNA stability and translation. Furthermore, m6A can exert an indirect influence on viral infections by orchestrating the expression of specific genes implicated in viral replication and pathogenesis. A profound understanding of the biological functions of m6A modification within the viral transcripts is pivotal for delineating their roles in viral replication and devising groundbreaking preventive strategies to combat viral infections. In this review, we endeavor to summarize the emerging roles of m6A RNA methylation in HBV infection and pathogenesis and engage in a comprehensive discussion regarding m6A functions related to viral replication processes.

## Regulation of HBV Life Cycle by m6A

**Fig. 3:**
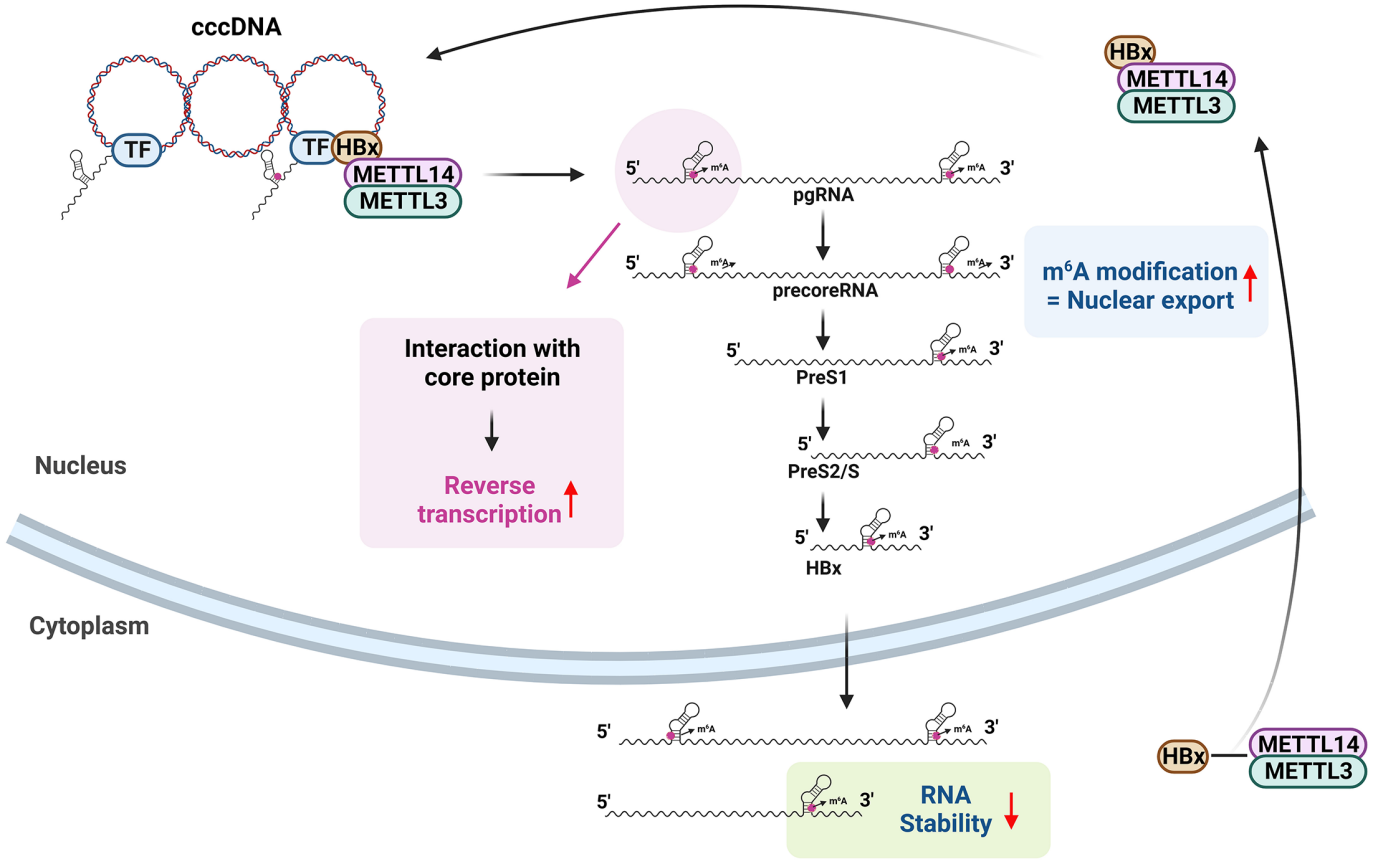
The role of m6A modification in regulating the HBV life cycle. In the context of the HBV life cycle, the HBV HBx protein plays a pivotal role in orchestrating m6A modification. Initially, HBx interacts with METTL3/14 in the cytosol, facilitating their subsequent nuclear import. During HBV transcription, HBx guides METTL3/14 to the HBV cccDNA, where they co-transcriptionally introduce m6A methylation into HBV transcripts. This m6A methylation occurs at a consensus m6A motif located within the lower stem of HBV epsilon structure. HBV pgRNA contains two of these motifs at its 5' and 3' termini due to terminal redundancy, whereas other viral transcripts have only one motif, typically in the 3' terminal sequence. Notably, the HBV 5' terminal m6A modification promotes the interaction with the core protein by affecting the region surrounding the priming site for reverse transcription initiation. This interaction, in turn, promotes the reverse transcription of HBV DNA from pgRNA. Conversely, the HBV 3' terminal m6A modification, present in all viral RNAs, decreases RNA stability by interacting with YTHDF2. Additionally, the reader proteins YTHDC1 and FMRP induce m6A methylation of HBV mRNA, subsequently enhancing translation. This intricate interplay of m6A modification mechanisms significantly impacts the regulation of the HBV life cycle.

HBV RNAs can undergo m6A methylation at several regions and this modification can have either direct or indirect impacts on the HBV life cycle through diverse pathways [29-34]. The m6A RNA modification predominantly occurs at the adenosine 1907 nucleotide (nt), located within the epsilon structure present across all HBV RNAs (Fig. 3) [29]. This epsilon structure is present at both the 5' and 3' ends of HBV pgRNA and precoreRNA due to terminal redundancy [1]. In contrast, other HBV mRNAs possess this m6A site only once in the 3' termini [1].

Imam et al. have demonstrated that the suppression of METTL3/14 triggers elevated stability and translation of HBV RNAs, and this outcome arises from m6A being mediated by the interaction of the m6A methylation at the lower stem of 3' epsilon of HBV RNAs with m6A readers of the YTHDF2/3 protein complex [29]. Conversely, the depletion of METTL3/14 negatively regulates HBV core-associated DNA levels. The mutation at the m6A site of the 3' end enhances HBV RNA and protein levels, yet leaves HBV core-associated DNA levels unaffected [29]. Introducing an m6A site mutation at the 5' end escalates HBV core-associated DNA levels but does not affect HBV RNA stability and protein levels [29]. In the case of m6A modification of the lower stem of 5' epsilon, it offers a more favorable RNA secondary structure in the context of the interaction with core proteins to promote nucleocapsid assembly [30]. This implies that m6A methylation of the 5' epsilon-hairpin enhances the reverse-transcription activity of pgRNA and subsequently synthesizes cccDNA. Apart from its roles in RNA stability and nucleocapsid assembly, the m6A modification at position 1907 nt can also impact the nuclear export of HBV transcripts [31]. When m6A levels in HBV RNA are reduced due to METTL3/14 depletion, this leads to an accumulation of HBV RNAs within the nucleus, suggesting that HBV RNAs bearing m6A modifications are facilitated in their export from the nucleus to the cytoplasm, thereby expediting the HBV life cycle. The m6A-mediated nuclear export of HBV RNA is responsible for interactions with m6A reader proteins like fragile X mental retardation protein (FMRP) and YTHDC1 [31]. Depletion of FMRP and YTHDC1 leads to the accumulation of HBV transcripts within the nuclear fraction, consequently reducing overall HBV replication. Hence, these results illustrate that m6A RNA methylation at 1907 nt can play multiple functions in the regulation of HBV replication and the specifics of these functions are intricately dependent on its position and interaction with various m6A reader proteins.

Furthermore, further variations of m6A modification have been identified within the region spanning from 1606 to 1809 nt. This region corresponds to both the 3' UTR of other viral mRNAs and the coding region of HBx mRNA [32]. By utilizing mutant m6A sites within the consensus DRACH motif from 1606 to 1809 nt, as well as a strategy involving the depletion of m6A methyltransferases (METTL3/14) and reader proteins (YTHDFs), it has been demonstrated that the m6A methylation at 1616 nt, located within the coding regions of HBx, reduces the levels of both HBx mRNA and protein [32]. However, other viral mRNA and protein expressions remain unaffected. This suggests that m6A modification within the HBx open reading frame (ORF) contributes to the downregulation of HBx protein expression, a phenomenon commonly observed in HBV transfections, transgenic mice, and HBV patient liver biopsy samples [1, 2, 35]. Overall, these findings illustrate the role of m6A modification at 1616 nt in subtly regulating the expressions of HBx mRNA and protein, which could be relevant to its potential involvement in establishing chronic hepatitis.

In a separate study, it was observed that the HBx, functioning as a transactivating protein, plays a role in introducing m6A modification co-transcriptionally into HBV transcripts (Fig. 3) [34]. HBV genomes lacking HBx expression fail to incorporate m6A modifications to HBV RNAs in both transfected and infected cells. However, introducing exogenous HBx protein restores the level of m6A modification in viral RNAs, except in the case of mutant HBx lacking the nuclear import signal. The authors have demonstrated that HBx guides METTL3 and 14 complexes to the HBV cccDNA, facilitating the addition of co-transcriptional m6A methylation to viral RNAs. Indeed, HBx interacts with METTL3/14, modestly enhancing their nuclear import. Inducing the nuclear import of METTL3/14 complex by HBx increases the levels of m6A modification in viral RNAs. This study sheds light on how a viral protein recruits m6A RNA methyltransferase machinery to intorduce m6A RNA modifications.

## Regulation of Innate Immunity by m6A

In recent years, the importance of m6A in immune regulation and antiviral responses has gained significant attention [36-39]. Specifically, the influence of m6A modifications on the strength and duration of innate immune response activation has been illuminated. Innate immunity operates with exquisite precision, delicately balancing between enhancement and suppression signals to ensure a potent antiviral response [40, 41]. Various viruses have been found to manipulate the innate immune response using m6A methylation, thereby contributing to the establishment of chronic infection. Investigating mechanisms through which these viruses suppress innate immune responses is crucial for overcoming viral infections.

Among the cytosolic pattern recognition receptor (PRR), the Retinoic acid-inducible gene I (RIG-I) signaling pathway constitutes one of the host's defense mechanisms crafted to counter viral infections [42, 43]. Within the cytosol, RIG-I binds to its ligands, pathogen-associated molecular pattern (PAMP) such as viral dsRNA and 5' triphosphate RNA. Detection of these ligand RNAs triggers polyubiquitination of RIG-I, initiating an interaction with mitochondrial antiviral signaling protein (MAVS) as the adaptor protein [40]. This interaction activates a cascade of downstream signaling pathways, leading to the phosphorylation of interferon regulatory factor-3 (IFR-3) and subsequent transcription of type I interferon (IFN) genes.

HBV adeptly evades the host's immune responses, even when the 5' epsilon structure of HBV pgRNA is recognized by RIG-I [42]. To evade the host innate immunity, the HBV HBx protein interferes with RIG-I/MAVS signals through the ubiquitination of the mitochondria-associated MAVS protein [44]. Furthermore, recent research has shed light on m6A methylation of HBV pgRNA as an additional level of immune suppression [45]. The m6A methylation at the 5' epsilon structure of pgRNA leads to a reduction in IRF-3 activation and IFN synthesis by inhibiting RIG-I recognition. In contrast, silencing METTL3/14 and mutating the m6A motif in the 5' epsilon region of pgRNA, thus abolishing m6A modification, significantly enhances RIG-I recognition. The underlying mechanism is connected to the interaction between pgRNA and m6A reader proteins YTHDF2 and YTHDF3. Depleting YTHDF2/3 activates the RIG-I-mediated innate immune response, leading to increased IFN synthesis during HBV replication. However, increasing levels of YTHDF2 and YTHDF3 expression disrupt the recognition of pgRNA by RIG-I. The interactions between YTHDF2 and YTHDF3 with pgRNA compete with RIG-I binding, suggesting that m6A methylation of HBV RNA could serve as molecular signatures distinguishing self from non-self-RNAs sensed by RIG-I. In light of this, HBV employs a camouflage strategy, mimicking cellular RNA through m6A methylation. Similar phenomena have been observed in positive strand RNA viruses, including severe acute respiratory syndrome-coronavirus-2 (SARS-CoV-2) and hepatitis C virus (HCV) [45-47]. This suggests that RNA viruses as well as DNA viruses can evade the recognition by host PRR using m6A methylation of their RNA genomes.

The innate immune response can also be influenced through the modulation of m6A methylation of host mRNA related to antiviral signals [48]. During HBV infection, there is an increase in the m6A methylation of phosphatase and tensin homolog (PTEN) mRNAs, resulting in reduced RNA stability and subsequently decreased protein expression [48]. PTEN, a tumor suppressor frequently diminished or mutated in various cancers [49], also plays a crucial role in IRF-3-mediated IFN synthesis [50]. PTEN promotes dephosphorylation of IRF-3 at the Ser97 residue, facilitating IRF-3 nuclear import and activating transcription of IFN mRNA. However, during HBV infection, the decreased stability of PTEN mRNA due to m6A methylation inhibits IRF-3 nuclear translocation and the transcription of IFN-beta mRNA [48]. Notably, the HBx protein enhances co-transcriptional m6A methylation of PTEN mRNA [34]. HBx accomplishes this process by recruiting m6A methyltransferases onto the PTEN chromosome locus. Thus, HBV infection evades the innate immunity by exploiting HBx-mediated m6A methylation of both its own RNA transcripts and host RNAs, ultimately contributing to the establishment of a chronic infection.

## Regulation of HBV-Mediated Liver Cancer by m6A

Persistent HBV infection can lead to liver fibrosis, thereby advancing the development of cirrhosis and hepatocellular carcinoma (HCC) [2]. Recently, the role of m6A methylation in HBV-related liver fibrosis, cirrhosis, and HCC has gained attention [51]. Zhao *et al*. found that the m6A patterns in cellular RNAs are associated with the liver fibrosis stage in patients with chronic HBV infection. Additionally, several HCC-related cellular RNAs have been identified to be m6A methylated and implicated in HBV-mediated HCC [48, 52]. The HBx protein increases m6A methylation of circular RNAs (circRNA)-ARL3, a factor which sequesters tumor-suppressive miRNA-1305 [52]. Increased m6A levels of circRNA-ARL3 by HBx enhance its interaction with m6A reader YTHDC1, promoting its biogenesis. Higher levels of circ-ARL3 inhibit miRNA-1305 function through sequestration, leading to HCC development.

Furthermore, HBV infection positively controls the phosphoinositide 3-kinase (PI3K)/AKT signaling pathway, a crucial intracellular signaling pathway that regulates cell proliferation and apoptosis [48]. As a lipid phosphatase, PTEN inhibits cell survival and apoptosis by disrupting of the PI3K/AKT pathway. As mentioned earlier, HBx increases m6A levels on PTEN mRNA, reducing RNA stability [34]. The activated PI3K/AKT signaling pathway resulting from reduced PTEN expression during HBV infection thereby causes uncontrolled cell growth [48, 53]. The decreased expression of PTEN caused by HBx expression in HBV infection could partially explain the oncogenic characteristics observed in transgenic mice expressing the HBx protein [54]. Therefore, the m6A-mediated reduction in PTEN protein production by HBx is one of the factors contributing to the development of cancer during HBV infection.

## Summary

In this review, we explored the recently discovered functions of m6A methylation in the context of HBV infection. m6A modification exhibits complex regulatory effects on the HBV life cycle depending on its location [29-33, 45]. Moreover, HBV infection orchestrates alteration in m6A methylation levels within host mRNA, which is a contributing factor in controlling chronic infection [48]. Ultimately, the regulation of HBV infection by m6A has significant implications for the development of liver disease, shedding light on previously undefined roles of m6A modification within the HBV infection. In addition, new functions for m6A modification in the epigenetic regulation of cellular RNAs, as well as viral RNAs, continue to emerge [20-28, 46, 55, 56]. Recent studies have emphasized the presence of m6A methylation in the RNA virus genome and RNA transcripts of DNA viruses. This m6A modification of viral transcripts has been demonstrated to influence different facets of viral life cycles and the progression of diseases. In addition to m6A methylation, RNA occurs in diverse RNA methylations, such as 5-methylcytosine (m5C) and 2-O-methylation, according to the different sites [57, 58]. These modifications also affect RNA splicing, stability, and translation. It is reported that m5C methylation occurs in the mRNA of HIV-1 and this methylation regulates mRNA translation and alternative splicing [22]. In the case of HBV, although other RNA methylations of the viral transcript have not yet been identified, it is believed if other RNA modifications can occur in HBV RNA, they may lead to addition of new concepts regarding HBV replication. Understanding the biological significance of m6A modification and other RNA methylation in viruses is crucial. It illuminates their roles in viral pathogenesis and opens avenues for innovative strategies in preventing viral infections.
